# XWL-1-48 exerts antitumor activity via targeting topoisomerase II and enhancing degradation of Mdm2 in human hepatocellular carcinoma

**DOI:** 10.1038/s41598-017-10577-7

**Published:** 2017-08-30

**Authors:** Yajie Wang, Hua Sun, Zhiyan Xiao, Dan Zhang, Xiuqi Bao, Ning Wei

**Affiliations:** 1 State Key Laboratory of Bioactive Substance and Function of Natural Medicines, Institute of Materia Medica, Chinese Academy of Medical Sciences and Peking Union Medical College, Beijing, China; 2 Institute of Chinese Materia Medica, China Academy of Chinese Medical Sciences, Beijing, China; 3 Division of Hematology-Oncology, Department of Medicine, University of Pittsburgh School of Medicine, Pittsburgh, PA USA; 4 Cancer Therapeutics Program, University of Pittsburgh Cancer Institute, University of Pittsburgh, Pittsburgh, PA USA

## Abstract

A novel podophyllotoxin derivative, XWL-1-48, was synthesized as an oral topoisomerase II inhibitor. kDNA decatenation assay indicated that XWL-1-48 significantly inhibited topoisomerase II activity in a concentration-dependent manner. Moreover, the cytotoxicity of XWL-1-48 is more potent than its congener GL331 and the IC_50_ values are from 0.34 ± 0.21 to 3.54 ± 0.54 µM in 10 cancer cell lines including KBV200 cells with P-gp overexpression. Noticeably, XWL-1-48 exerted potent antitumor activity in *in vitro* and *in vivo* human hepatocellular carcinoma (HCC) model. Further studies demonstrated that treatment of XWL-1-48 induced γ-H2AX and p-ATM expression, and further triggered DNA damage response through activation of ATM-p53-p21 and ATM-Chk2-Cdc25A pathways. Targeted inhibition of ATM by siRNA attenuated the ability of XWL-1-48 on inducing DNA damage. XWL-1-48 significantly suppressed Cyclin A and p-Cdk2 (Thr160) expression, increased p-Cdk2 (Thr14), led to inactivation of Cyclin A/Cdk2 complex, arrested cell cycle at S phase. Finally, XWL-1-48 elevated the ratio of Bax/Bcl2 and induced Fas and FasL, initiated mitochondria- and death receptor-mediated apoptosis pathway. Meanwhile, XWL-1-48 evidently enhanced degradation of Mdm2, blocked PI3K/Akt/Mdm2 pathway and suppressed HCC cell survival. Thus, XWL-1-48 may be a promising orally topoisomerase II inhibitor for treatment of HCC.

## Introduction

Hepatocellular carcinoma (HCC) is a primary malignant neoplasm derived from hepatocytes, accounting for about 80% of all types of liver cancer^1^. HCC is the fifth most common malignancy and the second leading cause of cancer-related deaths in the world^2, 3^. Noticeably, HCC has ranked the second common cancer in China^4^. HBV, HCV, alcohol consumption, and smoking as identified risk factors contribute to the incidence of HCC^5–8^. Despite advancements in surgical techniques improved overall survival (OS) rates among HCC patients, 5-year OS still low at 18%. Due to most patients cannot be treated by surgical resections or liver transplantation (LT), the survival rate of HCC patients is poor^9^. Moreover, recurrence rate of HCC is above 50% at 5 years after surgery^10^. Until now, chemotherapy is still the main strategy for system treatment for HCC. Therefore, there is an urgent to develop novel agents against HCC.

Topoisomerases II are a well-validated target for treatment of cancer. In eukaryotic cells, there are two isoforms of TopoII, α and β, create DNA double-stranded breaks (DSBs) to allow the passage of a second double-stranded DNA, correct topological DNA errors in replication, transcription, recombination, and chromosome condensation and decondensation^11^. These functions are helpful to genomic integrity. Based on the biological functions of topoisomerase II are important for ensuring genomic integrity, the ability to targeted inhibition of topoisomerase II and trigger DNA damage is a successful strategy for treatment of cancer^12^. Recently, researchers found that TOP2A (topoisomerase 2 gene) overexpression in hepatocellular carcinoma correlated with chemoresistance and shorter patients survival^13^. TopoisomeraseII poisons (e.g. etoposide, teniposide and VP16) are still frontline therapies for HCC^14^.

Podophyllotoxin, a traditional topoisomerase II inhibitor, exhibited potent antitumor activity. Due to serious side effects in clinic trails, podophyllotoxin fail to use as antitumor agent^15^. Noticeably, etoposide and teniposide, semisynthetic derivatives of podophyllotoxin, targeted inhibition of topoisomerase II activity and are currently used clinically for treatment of various types of cancer, including small-cell lung cancer, testicular carcinoma, lymphoma, and Kaposi’s sarcoma^16–18^. They appear to act by causing breaks in DNA via an interaction with DNA TopoII or by the formation of free radicals. Teniposide is more potent as regards the production of DNA damage and cytotoxicity. However, there is still several limitations such as poor water solubility, metabolic inactivation and development of drug resistance^19^. Therefore, researchers are trying to design and develop new derivatives of podophyllotoxin that could overcome these deficiencies. Herein, XWL-1-48, a new podophyllotoxin derivative was synthesized as oral topoisomerase II inhibitor. *In vitro* and *in vivo* antitumor activity of XWL-1-48 was evaluated in our HCC model. Interestingly, XWL-1-48 exhibited better watersolubility and more potent activity than VP16 and GL331 for oral administration. XWL-1-48 still killed multi-drug resistant cancer cells. What is more, the potential mechanism of XWL-1-48 against HCC is investigated.

## Results

### Effect of XWL-1-48 on topoisomerase II -mediated kDNA-decatenation

**Figure 1:**
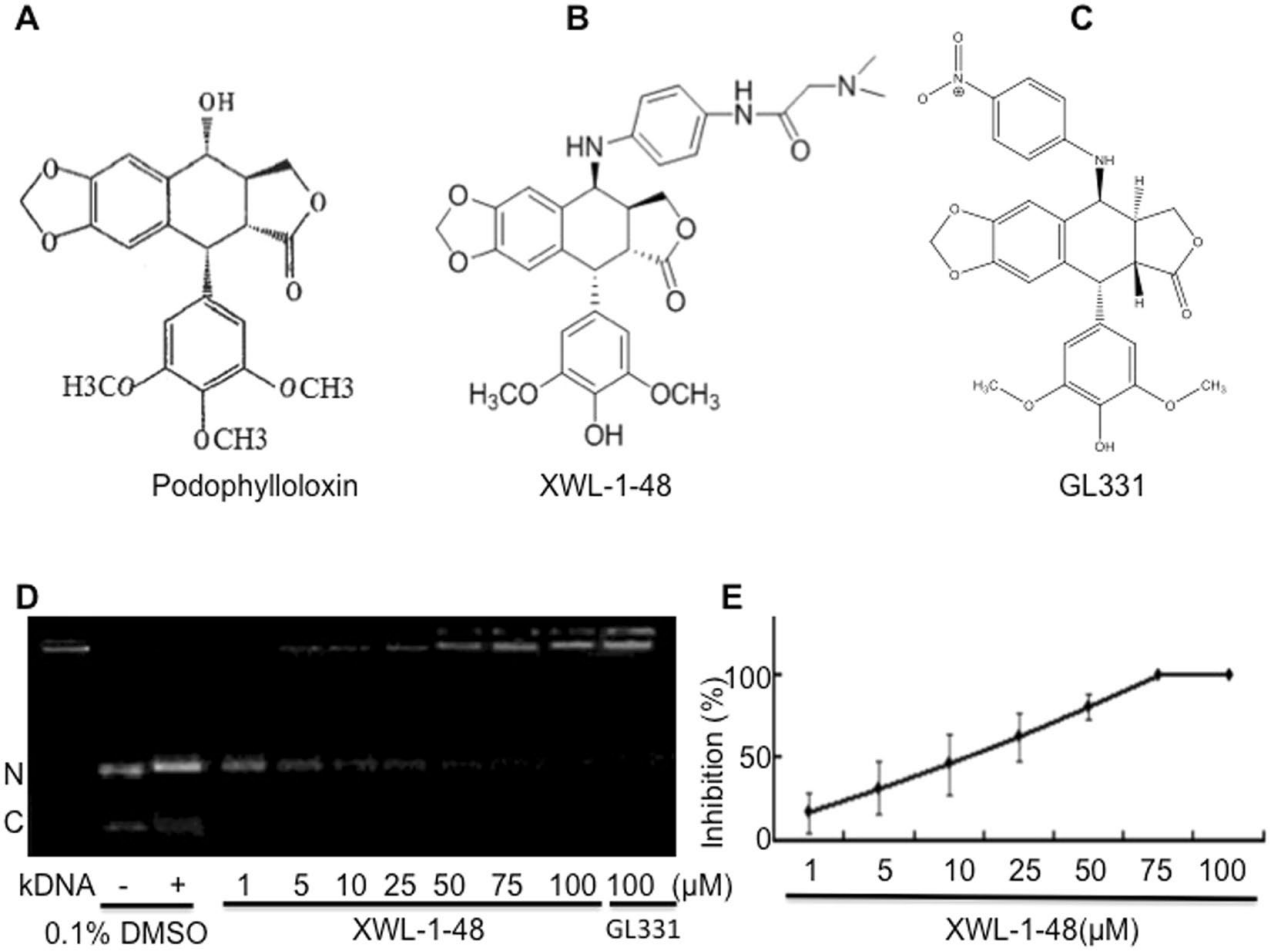
Effect of XWL-1-48 on topoisomerase II-mediated kDNA decatenation. The chemical structures of podophyllotoxin (**A**), XWL-1-48 (**B**) and GL331(**C**) are shown. (**D**,**E**) to determine the effect of XWL-1-48 on topoisomerase II activity, kDNA decatenation assay was performed as described in materials and methods. Data were shown as mean ± SD of three independent experiments. A representative of three independent experiments is shown (N: nicked decatenated DNA; C: circular decatenated DNA).

To determine the effect of XWL-1-48 on topoisomerase II activity, kDNA-decatenation assay was performed. As seen in Fig. 1D and E, kDNA-decatenation reaction indicated that XWL-1-48 significantly inhibited the topoisomerase II activity in a concentration-dependent manner. Noticeably, the ability of XWL-1-48 to suppress topoisomerase II activity is more potent than that of GL331.

### Effect of XWL-1-48 on proliferation of human cancer cells

**Figure 2:**
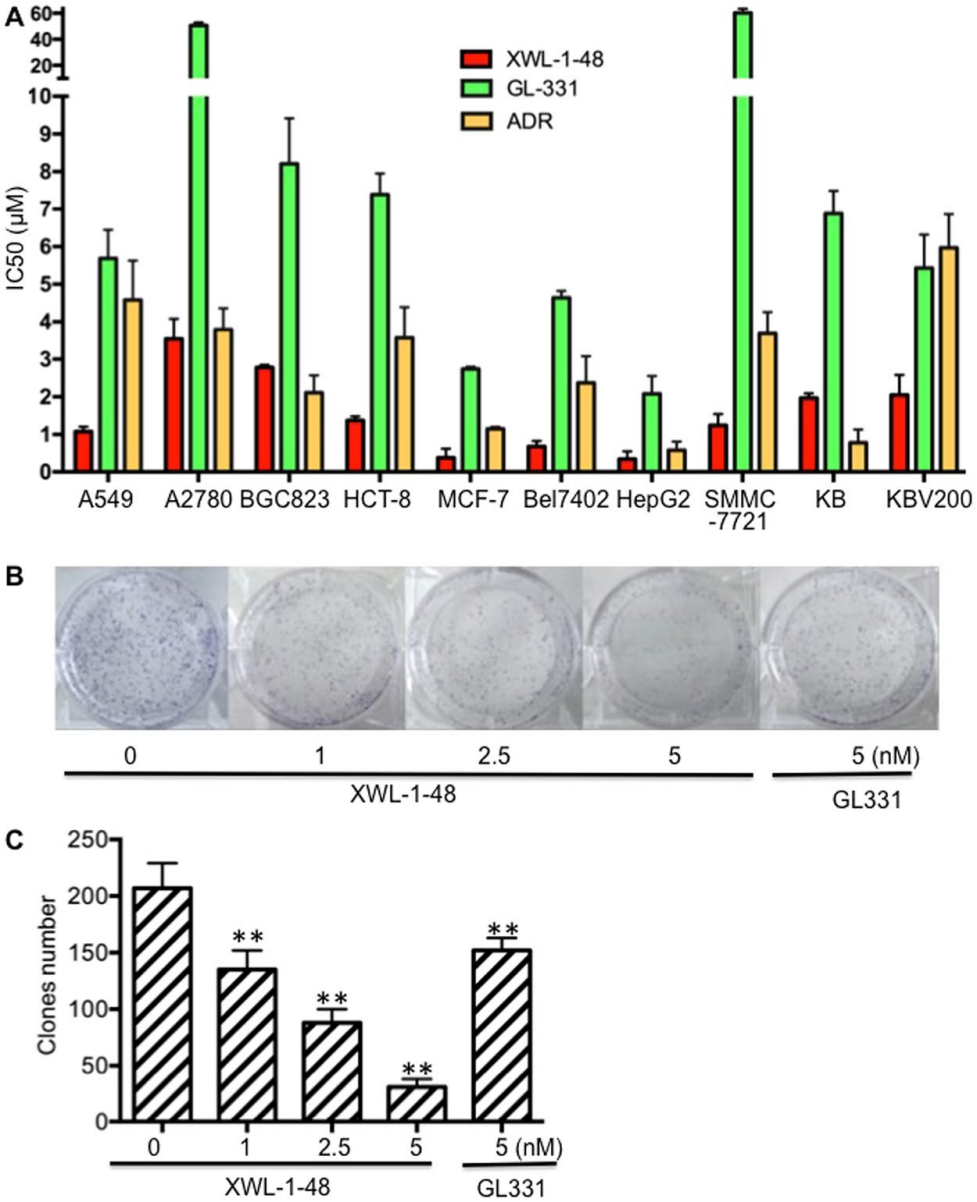
Effect of XWL-1-48 on growth of human cancer cells *in vitro*. (**A**) The cytotoxic effect of XWL-1-48 on growth of human cancer cells was determined by MTT assay. IC_50_ was calculated by Prism6.0 software. The results represent the mean ± SD of three independent experiments each done in duplicate. (**B**) HepG2 cells were seeded in a 6-well plate, and incubated with XWL-1-48 (1, 2.5, 5 nM) or GL331 (5 nM) for 24 h. After 10–14 days, colonies (>50 cells) were fixed and manually counted. A representative of three experiments is shown. (**C**) Data were shown as mean ± SD of three independent experiments. ^**^ *p* < 0.01 *vs*. control.

To evaluate cytotoxic activity of XWL-1-48, a wide range of human cancer cell panel (including A549, A2780, HCT-8, MCF-7, Bel7402, HepG2, SMMC-7721, KB and KBV200; Fig. 2A) were incubated with different concentrations of XWL-1-48, GL331 and ADR for 72 h. Cell proliferation was determined by the MTT assay. As shown in Fig. 2A, XWL-1-48 exhibited potent cytotoxicity, the IC_50_ values range from 0.34 ± 0.21 to 3.53 ± 0.54 μM in various types of cancer cells, including HCC, lung cancer, breast cancer, colorectal cancer, and so on. Interestingly, the cytotoxicity of XWL-1-48 is more potent than that of GL331 in 10 human cancer cell lines. Due to overexpression of P-gp, KBV200 cells displayed MDR phenotype. Herein, the IC_50_ of XWL-1-48 in KB and KBV200 cells is 1.97 ± 0.12 and 2.05 ± 0.53 μM, respectively. The similar suppression of XWL-1-48 on growth of KBV200 and KB cells suggested that it had good anti-MDR activity. In addition to the MTT assay, we utilized the clonogenic assay to determine the effect of XWL-1-48 on HepG2 clonogenic growth (Fig. 2B and C). XWL-1-48 significantly decreased colony number in a dose-dependent manner with low IC_50_ values (~2.5 nM).

### XWL-1-48 induces S phase arrest in HCC

**Figure 3:**
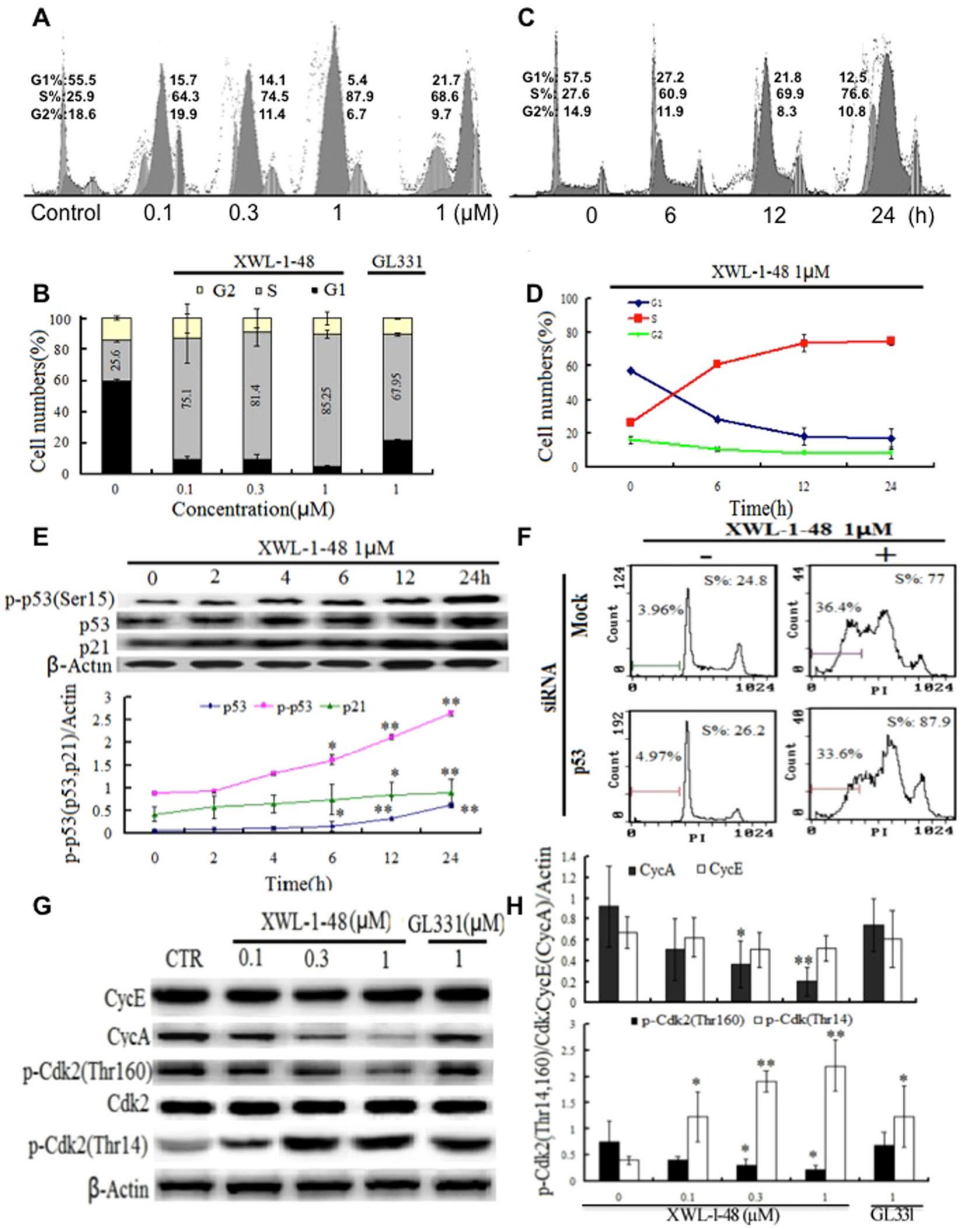
Effect of XWL-1-48 on cell cycle in HCC. (**A**) HepG2 cells were treated with XWL-1-48 (0.1, 0.3, 1 µM) and GL331 (1 µM) for 24 h, then cells were harvested, fixed and stained with PI for flow cytometry. (**B**) Data were shown as mean ± SD of three independent experiments. (**C**,**D**) HepG2 cells were incubated with XWL-1-48 (1 µM) for indicated time point, and cell cycle distribution was measured by flow cytometry. (**E**) HepG2 cells were treated by XWL-1-48 for 0, 2, 4, 6, 12, 24 h. The expression of p-p53 (ser15), p53 and p21 was determined by western blot analysis. A representative of image and curve from three independent experiments is shown. (**F**) Targeted inhibition of p53 using siRNA for 24 h, further treated by XWL-1-48 for an additional 24 h, then cell cycle were detected by flow cytometry. (**G**,**H**) after exposure to XWL-1-48 (0.1, 0.3, 1 µM) and GL331 (1 µM) for 24 h, CycE, CycA, p-CDK2 (thr12, thr160) and CDK were determined by western blot analysis. A representative result of three experiments is shown. *p < 0.05, **p < 0.01 vs. control.

Based on potent anti-proliferative activity, effect of XWL-1-48 on cell cycle was analyzed by FCM. After treated by XWL-1-48 for 24 h, S-phase population of HepG2 cells was significantly increased in a concentration-dependent manner (Fig. 3A and B). Stronger effect of XWL-1-48 than GL331 on cell cycle was observed. Meanwhile, treatment of XWL-1-48 for 0, 6, 12, 24 h, cell cycle was analyzed. S phase was evidently increased when the cells were only exposed to XWL-1-48 for 6 h (Fig. 3C and D). p53 protein and downstream p21 protein are the most important G1/S phase checkpoint proteins involved^20^. Given the ability of XWL-1-48 on blocking S-phase, p53, p21 were determined by immunoblot analysis. Treatment of XWL-1-48 resulted in an increase of p-p53 and p21 in a time-dependent manner (Fig. 3E). Meanwhile, knockdown p53 using siRNA did not affect the ability of XWL-1-48 on inducing apoptosis and cell cycle arrest (Fig. 3F). Cyclin A(E)/CDK2 complex plays an important role in regulation of S-phase arrest. Effect of XWL-1-48 on expression of cyclin A, E, p-CDK2 and CDK2 were also studied. Elevation of cyclin A and activation of CDK2 was response to XWL-1-48 treatment (Fig. 3G). Thus, the alteration of p53/p21 and cyclinA/CDK2 complex by XWL-1-48 may be responsible to the blockage of cell cycle at S-phase.

### XWL-1-48 induce intrinsic and extrinsic apoptosis of HCC

**Figure 4:**
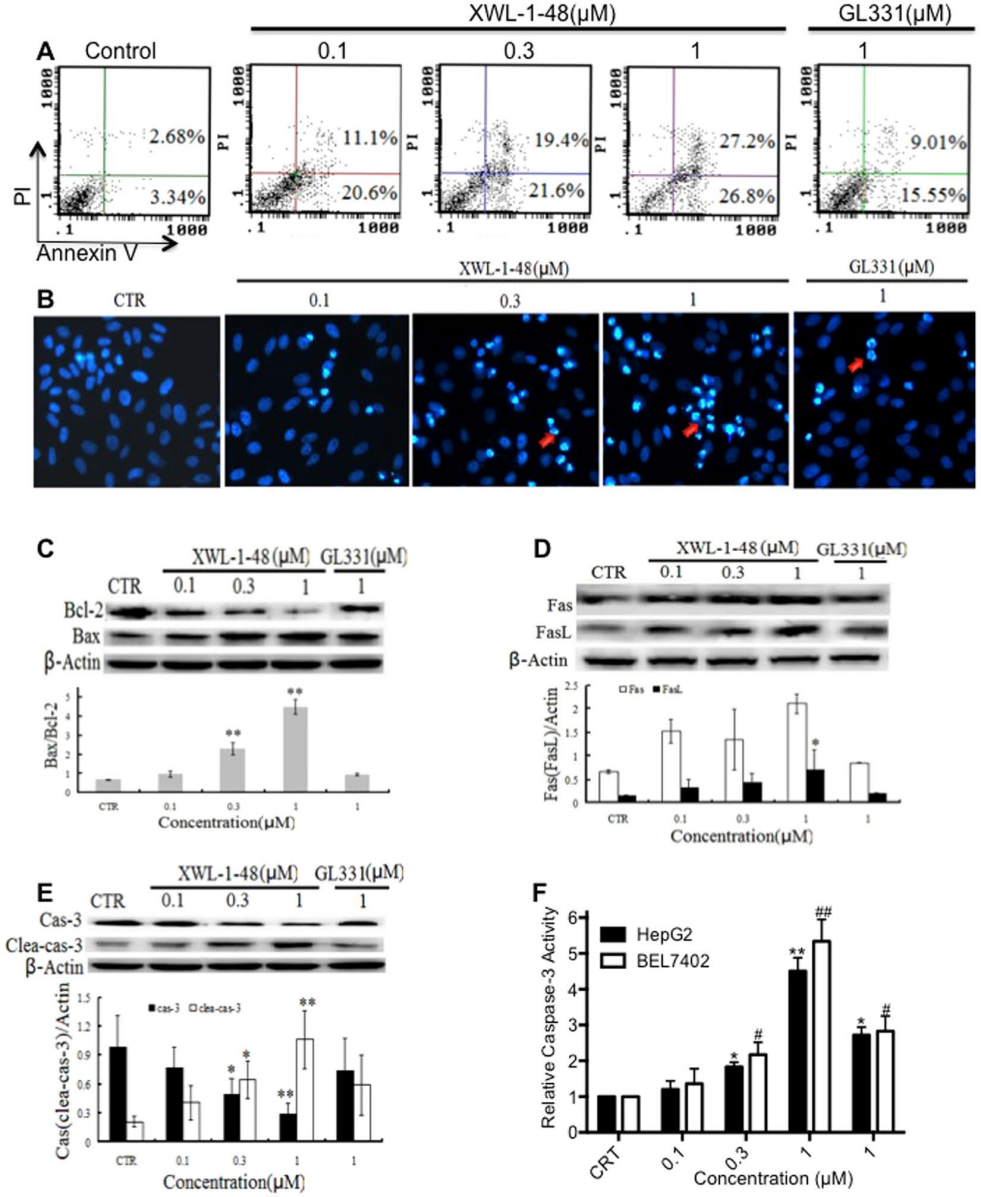
Effect of XWL-1-48 on cellular apoptosis of HCC. (**A**) Treatment with XWL-1-48 (0.1, 0.3, 1 µM) and GL331 (1 µM) for 24 h, early and late apoptotic cells were detected by flow cytometry. A representative of three independent experiments is shown. (**B**) Apoptosis of HepG2 cells was detected by DAPI staining. HepG2 cells were treated by XWL-1-48 or GL331 for 24 h, then the cells harvested, fixed and stained with 4, 6-diamidino-2-phenylindole (DAPI). (**C**,**D** and **E**) HepG2 cells were incubated with XWL-1-48 (0.1, 0.3, 1 µM) and GL331 (1 µM) for 24 h, expression of Bcl2, Bax, Fas, Fas L, cleave caspase-3 and caspase-3 were determined by western blot analysis. A representative result is shown from at least three independent experiments. (**F**) In addition, effect of XWL-1-48 on caspase-3 activity was measured by Caspase-3 colorimetric assay. Data were shown as mean ± SD of three independent experiments, *p < 0.05, **p < 0.01 *vs*. control group, respectively.

Based on evidently increase of sub-G1 population, it suggests that XWL-1-48 could induce cellular apoptosis in HCC. Accordingly, effect of WXL-1-48 on inducing apoptosis of HepG2 cells was determined by using Annexin V/PI and DAPI staining. As shown in Fig. 4A, WXL-1-48 resulted in an increase of early and late apoptotic cells in a dose-dependent manner. The ability of XWL-1-48 on inducing apoptosis is stronger than that of GL331. Meanwhile, DAPI staining was performed to observe the morphological changes of apoptosis. HepG2 cells with normal morphology were seen in control group, whereas HepG2 cells with fragmented chromatin and apoptotic bodies were observed in XWL-1-48-treated group (Fig. 4B). These results suggest that XWL-1-48 lead to marked apoptotic morphological changes in HepG2 cells.

Bcl-2 family proteins play a major role in the biological process of mitochondria-mediated intrinsic apoptosis^21^. The expression of pro-apoptotic protein Bax and anti-apoptotic protein Bcl-2 was determined by immunoblot analysis. As shown in Fig. 4C, treatment of XWL-1-48 significantly increased Bax expression and reduced Bcl-2 expression. Thus, the ratio of Bax and Bcl-2 was evidently elevated. The results suggest that XWL-1-48-mediated apoptosis is closely related to the mitochondrial pathway. In the other hand, Fas belongs to the TNF receptors superfamily which initiates apoptosis by recruiting a number of adaptor, signaling, and effector proteins^22^. Herein, effect of XWL-1-48 on Fas and FasL were also investigated. Treatment of XWL-1-48 significantly induced Fas and FasL expression in HepG2 cells (Fig. 4D). As a key effector in the process of apoptosis, capase-3 is well studied. The effect of XWL-1-48 on the activation of caspse-3 was measured using immunoblot and caspase-3 activity assay. Similar response of XWL-1-48 on activation of caspase-3 was observed (Fig. 4E and F). XWL-1-48 effectively activated caspase-3 in a dose-dependent manner in HepG2 and Bel7402 cells. Taken together, our results suggest that the apoptosis induced by XWL-1-48 is through mitochondria-mediated intrinsic and Fas/FasL-mediated extrinsic apoptosis pathway in HCC.

### Effect of XWL-1-48 on DNA damage response

**Figure 5:**
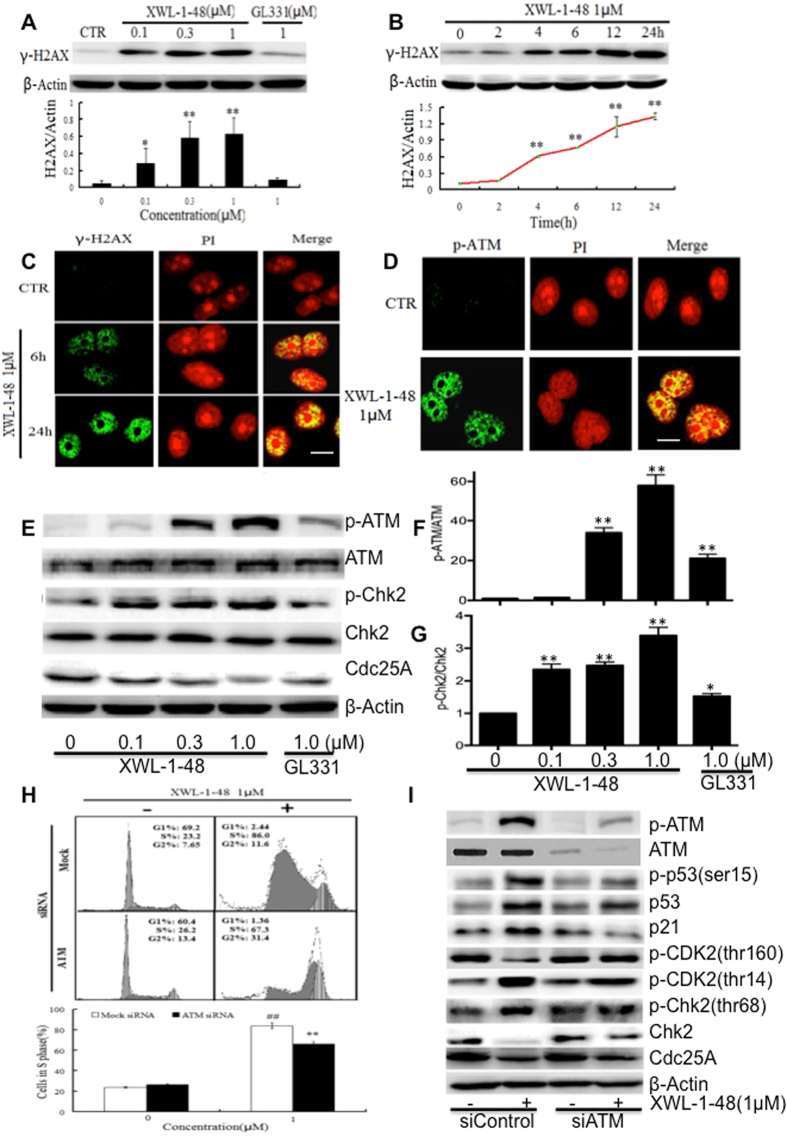
Effect of XWL-1-48 on DNA damage response in HCC. (**A**) HepG2 cells were treated with XWL-1-48 or GL331 for 24 h, induction of γ-H2AX was determined by western blot. (**B**) HepG2 cells were incubated with XWL-1-48 (1 µM) for indicated time point, γ-H2AX expression was measured. (**C**,**D**) Immunofluorescent staining of γ-H2AX and p-ATM in HepG2 cells. Cells were incubated in 6-well plate overnight and then exposed to XWL-1-48 (1 µM) for 6, 24 h. Cells were incubated with γ-H2AX or p-ATM antibody followed by incubation of secondary anti-rabbit IgG-FITC antibody (green). The nucleus was stained with PI (red). Images were captured using fluorescence microscope. Scale bar, 25 µm. (**E**,**F** and **G**) XWL-1-48 induced DDR by triggering ATM-related signaling pathways in HepG2 cells. Expression levels of ATM, p-ATM, p-Cdk2 (Thr160 and Thr14), Cdk2, p-Chk2 (Thr68) and Chk2 were analyzed. Data were shown as mean ± SD of three independent experiments. *p < 0.05, **p < 0.01 vs. control, respectively; (**H**,**I**) Targeted inhibition of ATM using siRNA for 24 h, then further incubated with XWL-1-48 for additional 24 h, cell cycle and S phase related proteins (including p53, p21, CDK2, Chk2 and Cdc25A) were determined by flow cytometry and immunoblot, respectively.

As a novel topoisomerase II poison, effect of XWL-1-48 on DNA damage response was also evaluated. HepG2 cells were incubated with XWL-1-48 for 24 h, γ-H2AX and p-ATM, ATM, p-Chk2 and Chk2 was determined by immunoblot analysis. As shown in Fig. 5A, treatment of XWL-1-48 significantly induced γ-H2AX expression in a concentration-dependent manner. In addition, XWL-1-48 led to an increase of γ-H2AX in a time-dependent manner (Fig. 5B). The expression of γ-H2AX was evidently increased when the cells were only exposure to XWL-1-48 for 4 h. Elevation of γ-H2AX in nucleus was observed using immunofluorescence microscopy (Fig. 5C). Taken together, XWL-1-48 effectively induced γ-H2AX in a dose- and time-dependent manner. We further measured the effect of XWL-1-48 on activation of ATM. 0.3 μM of XWL-1-48 significantly induced p-ATM expression. Similar response to XWL-1-48 on ATM activation was detected by using immunofluorescence microscopy. As shown in Fig. 5D, treatment of 1 μM XWL-1-48 potently enhanced expression of p-ATM in nucleus. Meanwhile, downstream mediators of ATM pathway were determined, including p-Chk2 and Cdc25A. XWL-1-48 caused activation of Chk2 in a concentration-dependent manner (Fig. 5E,F and G). Furthermore, we found that inhibition of ATM by siRNA partially reverse the ability of XWL-1-48 on cell cycle. Knockdown ATM abrogated the effect of XWL-1-48 on activation of ATM/Chk2/Cdc25A and ATM/p53/p21 pathways. Taken together, our findings demonstrated that XWL-1-48 triggered evidently DNA damage response, activated ATM/Chk2/Cdc25A and ATM/p53/p21 pathways, and further regulated cell cycle and cellular apoptosis.

### Effect of XWL-1-48 on PI3K/Akt/Mdm2 pathway

**Figure 6:**
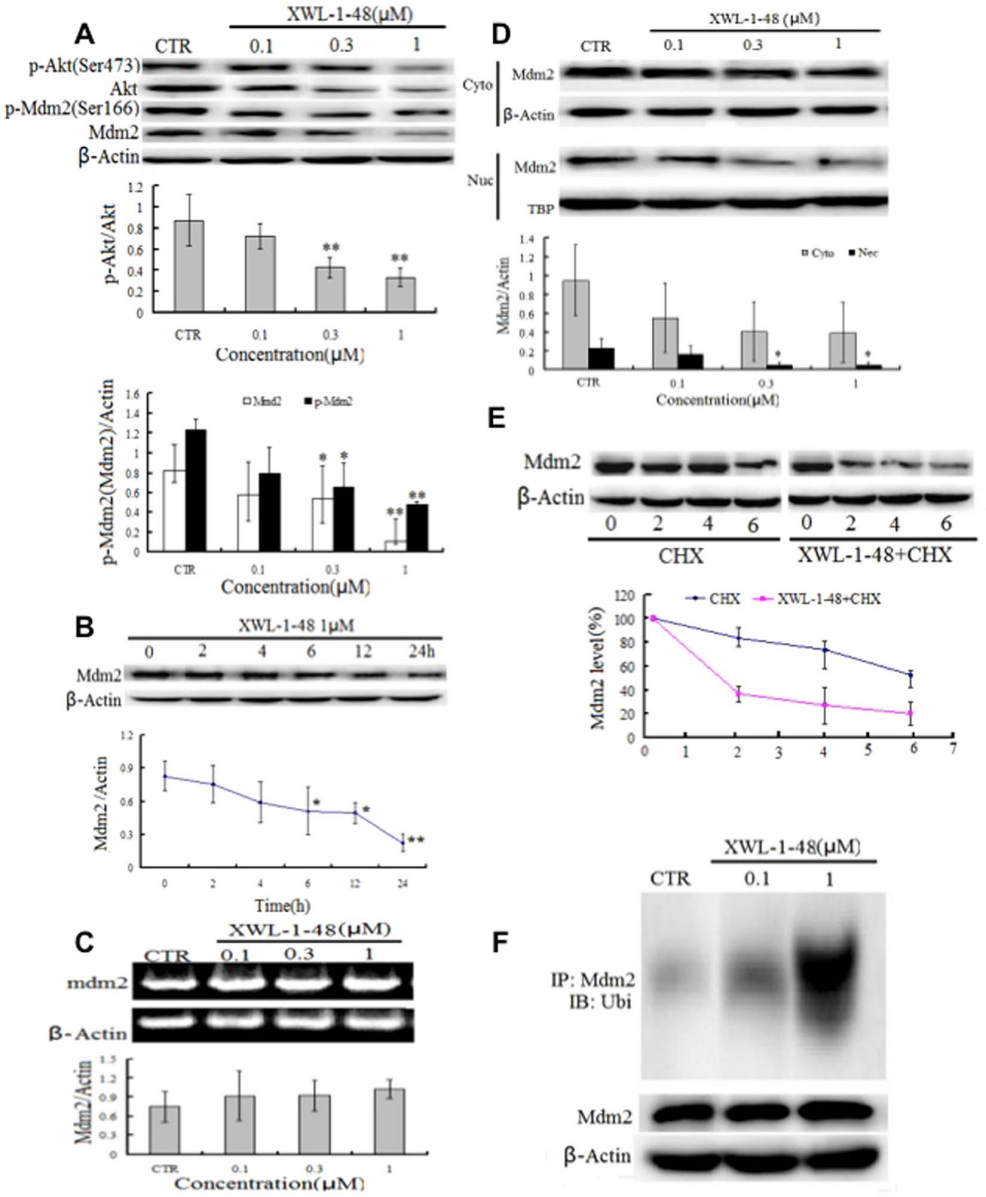
Effect of XWL-1-48 on PI3K/Akt/Mdm2 pathway. (**A**) HepG2 cells were treated by XWL-1-48 (0.1, 0.3, 1 μM) for 24 h, protein expression of p-Akt, Akt, p-Mdm2 and Mdm2 were determined by Western bolt analysis. The changes of p-Akt/Akt and p-ATM/ATM were quantified using Image J software. A representative result is shown from at least three independent experiments. (**B**) HepG2 cells were exposure to 1 μM of XWL-1-48 for 0, 2, 4, 6 12 and 24 h, protein expression level of Mdm2 was determined using immunoblot, and further quantified by image J software. (**C**) After treated with XWL-1-48 (0.1, 0.3, 1 μM) for 24 h, Cells were harvested. The mRNA level of Mdm2 was analyzed by RT-PCR. (**D**) After incubated with XWL-1-48 for 24 h, the expression of Mdm2 in cytoplasm and nuclear were detected. (**E**) HepG2 cells were exposed to 100 μg/ml cycloheximide (CHX, known as protein synthesis inhibitor) with or without XWL-1-48 (1 μM) for 2, 4, 6 h to block protein synthesis. The cells were collected for Western blot analysis. (**F**) Cells were treated for 48 h with or without XWL-1-48 (1 μM). Cell lysates were immunoprecipitated with 1 μg of anti-Mdm2 polyclonal antibody, followed by Western blotting with a monoclonal anti-ubiquitin antibody. A representative result is shown from at least three independent experiments. *p < 0.05, **p < 0.01 vs. control, respectively.

p53 is activated in response to DNA damage, lead to induction of apoptosis and inhibition of cancer cell growth^23^. Mdm2 play a key role in p53-mediated signaling pathway. Interestingly, there is cross-talk between Akt and the p53 pathway. Under DNA damage condition leading to an irreversible apoptotic event, activation of p53 may contribute to apoptosis by inhibition of Akt^24^. Meanwhile, in the presence of appropriate survival signals, Akt activation may lead to Mdm2 phosphorylation. Therefore, we investigated effect of XWL-1-48 on PI3K/Akt/Mdm2 pathway. As seen in Fig. 6A, XWL-1-48 significantly suppressed activation of Akt and decreased p-Mdm2 and total Mdm2 expression. Similar inhibitory effect of LY294002, a known pan-PI3K inhibitor, on PI3K/Akt/Mdm2 pathway was observed (data not shown). HepG2 cells were incubated with XWL-1-48 for 0, 2, 6, 12 and 24 h, Mdm2 expression was determined by immunoblot. XWL-1-48-treatment reduced Mdm2 in a time-dependent manner. After exposure to XWL-1-48 for 6 h, expression of Mdm2 was significantly decreased in HepG2 cells (Fig. 6B). The expression of Mdm2 in cytoplasmic and nucleus was also detected. Treatment with XWL-1-48 led to decrease of Mdm2 in cytoplasmic and nucleus (Fig. 6D). Meanwhile, effect of XWL-1-48 on transcription level of Mdm2 was measured using RT-PCR. Our result showed that XWL-1-48 did not affect mRNA expression level of Mdm2 (Fig. 6C). Above results indicated that XWL-1-48 significantly inhibited Mdm2 expression but did not affect the transcriptional level of Mdm2. It suggests that XWL-1-48 might affect the degradation of Mdm2. To test this possibility, we determined the half-life of Mdm2 protein by the introduction of cycloheximide (CHX), a known protein synthesis inhibitor. In presence of XWL-1-48, the Mdm2 protein had a shorter half-life (1.8 h) than that (6 h) of the control (Fig. 6E). By the way, we also determined the ubiquitination level of Mdm2 in HepG2 cells with or without XWL-1-48. As shown in Fig. 6F, treatment with XWL-1-48 increased the ubiquitination of Mdm2. These results suggested that XWL-1-48 could prompt Mdm2 degradation through enhancing the ubiquitination of Mdm2.

### *In vivo* biological activity of XWL-1-48 on the growth of HCC

**Figure 7:**
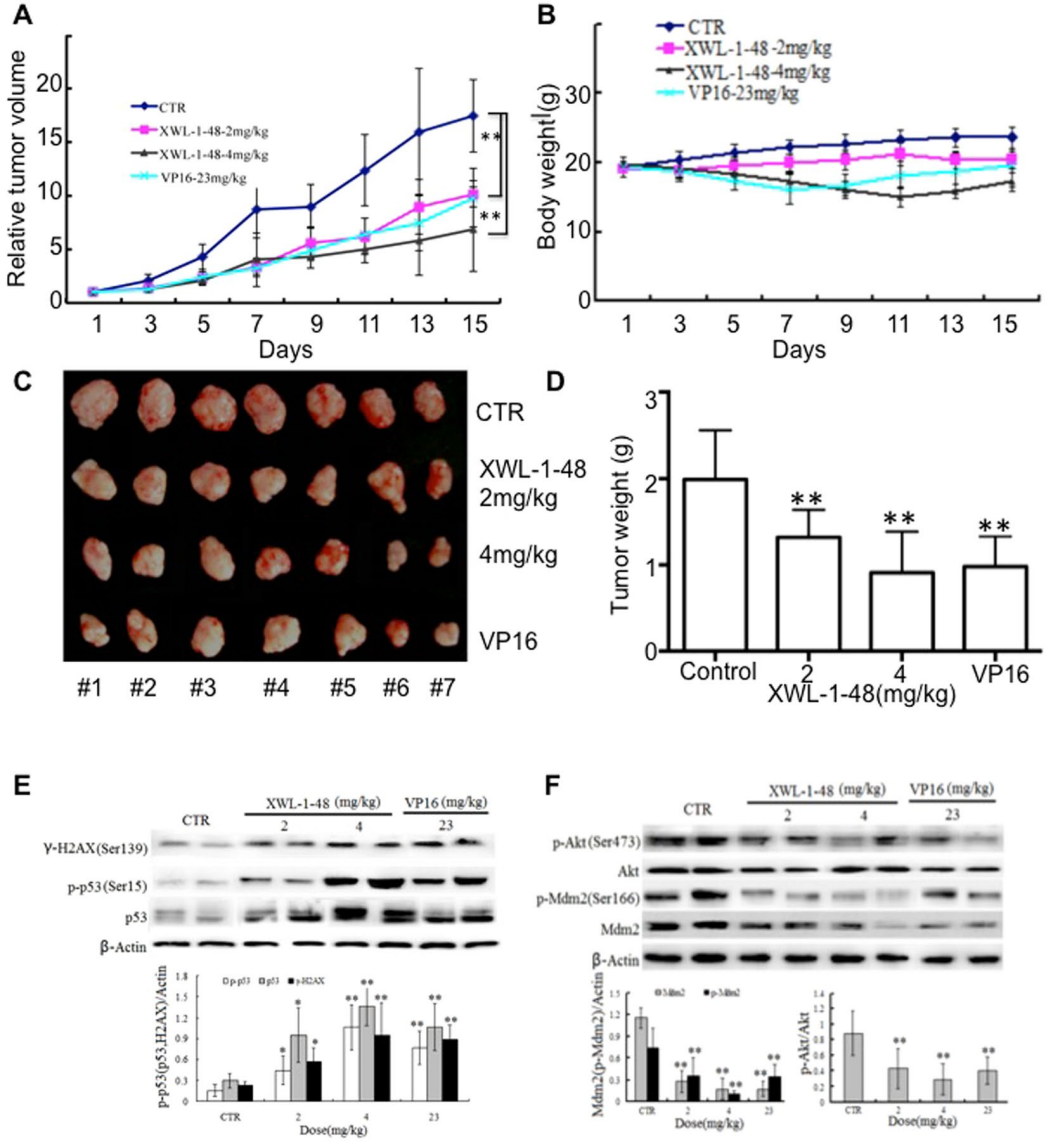
Effect of XWL-1-48 on growth of HCC *in vivo*. (**A**,**B**) Athymic nude mice bearing HepG2 tumor xenografts were orally administered every other day vehicle control (sterile normal saline), XWL-1-48 (2 and 4 mg/kg), and VP16 (23 mg/kg) for 15 days. Relative Tumor Volume and body weight were measured every other day. (**C**) The picture showed the tumor size of HepG2 xenograft at the end of the experiment. (**D**) Tumor tissues were weighed and further inhibitory rate of tumor weight were calculated. Data are mean ± SD of the tumor weight for each group of 7 experimental animals. (**E**,**F**) Protein expression in xenografts was determined after 15-day of XWL-1-48 or VP16 treatment. Tumors were harvested 2 h after the last dose. γ-H2AX, p-p53, p53; p-Akt, Akt, p-Mdm2 and total Mdm2 were analyzed by immunoblot analysis.

Given that the potent cytotoxic activity of XWL-1-48 against HCC cells, we further evaluated *in vivo* antitumor activity of XWL-1-48 using mice bearing HepG2 xenograft. Mice were orally administered different doses of XWL-1-48 (2 and 4 mg/kg) every other day for 15 days. At the end of the treatment period, tumor volume was decreased 34.2% and 58.7%, respectively, as compared with vehicle-treated mice (Fig. 7A). Inhibition of high dose XWL-1-48 on tumor growth is more potent than that of positive control VP16 (23 mg/kg). No gross toxicitiy in low-dose XWL-1-48 group was evident as determined by body weight, whereas decrease of body weight in XWL-1-48 (4 mg/kg) and VP16-treated group was observed (Fig. 7B). It suggests that high dose of XWL-1-48 exhibits some toxicity in our HepG2 xgenograft mice. Meanwhile, the tumor weight was also measured at the end of experiment. Inhibitory rate of tumor weight was 28.0% and 56.1%, respectively (Fig. 7C and D). Tumor tissue was obtained on day 15 and protein expression was determined by westernblot analysis. Administration of XWL-1-48 to the mice resulted in a dose-dependent activation of H2AX and p53 in the xenograft tumors (Fig. 7E). In addition, expression levels of key protein in PI3K/Akt/Mdm2 pathways were investigated. We observed potent suppression of p-Akt, p-Mdm2 and Mdm2, which correlated with our *in vitro* results (Fig. 7F).

## Discussion

In the present study, a novel podophyllotoxin derivative was synthesized as orally topoisomerase II inhibitor. kDNA decatenation assay indicated that XWL-1-48 potently inhibited topoisomerase II activity. XWL-1-48 exhibited strongly antitumor activity against 10 cancer cell lines, including lung cancer, liver cancer, breast cancer, colorectal cancer, and so on. Particularly, HCC cells (HepG2, Bel7402 and SMMC-7721) are more sensitive to XWL-1-48 than other types of cancer cells. The IC_50_ of XWL-1-48 in HCC cells is lower than 1 micromolar. What is more, XWL-1-48 displayed anti-MDR activity in KBV200 cells which overexpression of p-gp and resistant to anticancer agents. Noticeably, potent antitumor efficacy of XWL-1-48 in HCC xenograft by orally administration was observed. Meanwhile, the precise mechanism of XWL-1-48 against HCC is well studied.

As a new topoisomerase II poison, we further evaluated the ability of XWL-1-48 on cell cycle, apoptosis and DNA damage. Double stranded breaks (DSBs) occurs when DNA damage, it is always followed by the phosphorylation of γ-H2AX. It can be phosphorylated by kinases such as ATM and ATR^25^. This phosphorylated protein, γ-H2AX, is the first step in recruiting and localizing DNA repair proteins. DSBs can be induced by mechanisms such as cytotoxic agents (topoisomerase II inhibitor). Thus, γ-H2AX can be used as a biomarker for DNA damage. In the present study, induction of γ-H2AX in nucleus was observed using immunofluorescence microscopy. Further experiments showed that XWL-1-48 effectively induced γ-H2AX in a dose- and time-dependent manner. Meanwhile, XWL-1-48 evidently induced p-ATM in a dose-dependent manner, and then activated ATM/Chk2/Cdc25A pathway. Targeted inhibition of ATM caused reversal the ability of XWL-1-48 on inducing DNA damage response. Noticeably, when Chk2 triggers events that ultimately repair a DSB, the phosphorylation of the well-known p53 event also occurs^26^. Herein, XWL-1-48 significantly activated p53 in a time-dependent manner. Treatment of XWL-1-48 led to increase of p21, cyclin A/CDK2 and finally arrested cell cycle at S phase. In addition, apoptosis is a main cytotoxic mechanism of chemotherapeutic agents including topoisomerase II inhibitors. p53 protein can induce the expression of numerous apoptotic genes that can contribute to the activation of both death-receptor and mitochondrial apoptosis pathways. In this study, we found that XWL-1-48 significantly elevated the ratio of Bax/Bcl2, induced Fas and FasL, and finally initiated mitochondrial and death receptor-mediated apoptosis.

The p53 tumor suppressor plays an important role in cancer therapy, with p53-mediated cell growth arrest and/or apoptosis being major mechanisms of action for most of cancer chemotherapeutic agents, including topoisomerase II inhibitors^27^. p53 is primarily regulated by Mdm2 which binds p53 at its transactivation domain blocking p53-mediated transcriptional regulation and simultaneously induces p53 degradation^28–30^. The Mdm2 is a target gene for direct transcriptional activation by p53, and Mdm2 protein is a negative regulator of p53. Disrupting Mdm2-p53 interaction with resultant increase in p53 induces cancer cell cycle arrest and apoptosis. Therefore, the Mdm2-p53 interaction is a promising target for cancer therapy. What is more, the negative regulation of p53 by Mdm2 may limit the magnitude of p53 activation by DNA damaging agents, thereby limiting their therapeutic effectiveness^31^. Recently, researchers demonstrated that PI3K/Akt pathway inhibits p53-mediated transcription and apoptosis. Activation of Akt enhances the ubiquitination-promoting function of Mdm2 by phosphorylation of Ser^186^, which results in reduction of p53 protein^32^. Given that the effect of XWL-1-48 on induction of p-p53 and p53, we further investigated the effect of PI3K/Akt/Mdm2 pathway. Herein, we confirmed by Western blot that treatment of XWL-1-48 resulted in reduced p-Akt, p-Mdm2 and Mdm2. Inhibition of Mdm2 in a dose- and time-dependent manner was observed in XWL-1-48- treatment HepG2 cells. Further studies demonstrated that XWL-1-48 prompted degradation of Mdm2 through ubiquantion and decreased the half-life of Mdm2.

**Figure 8:**
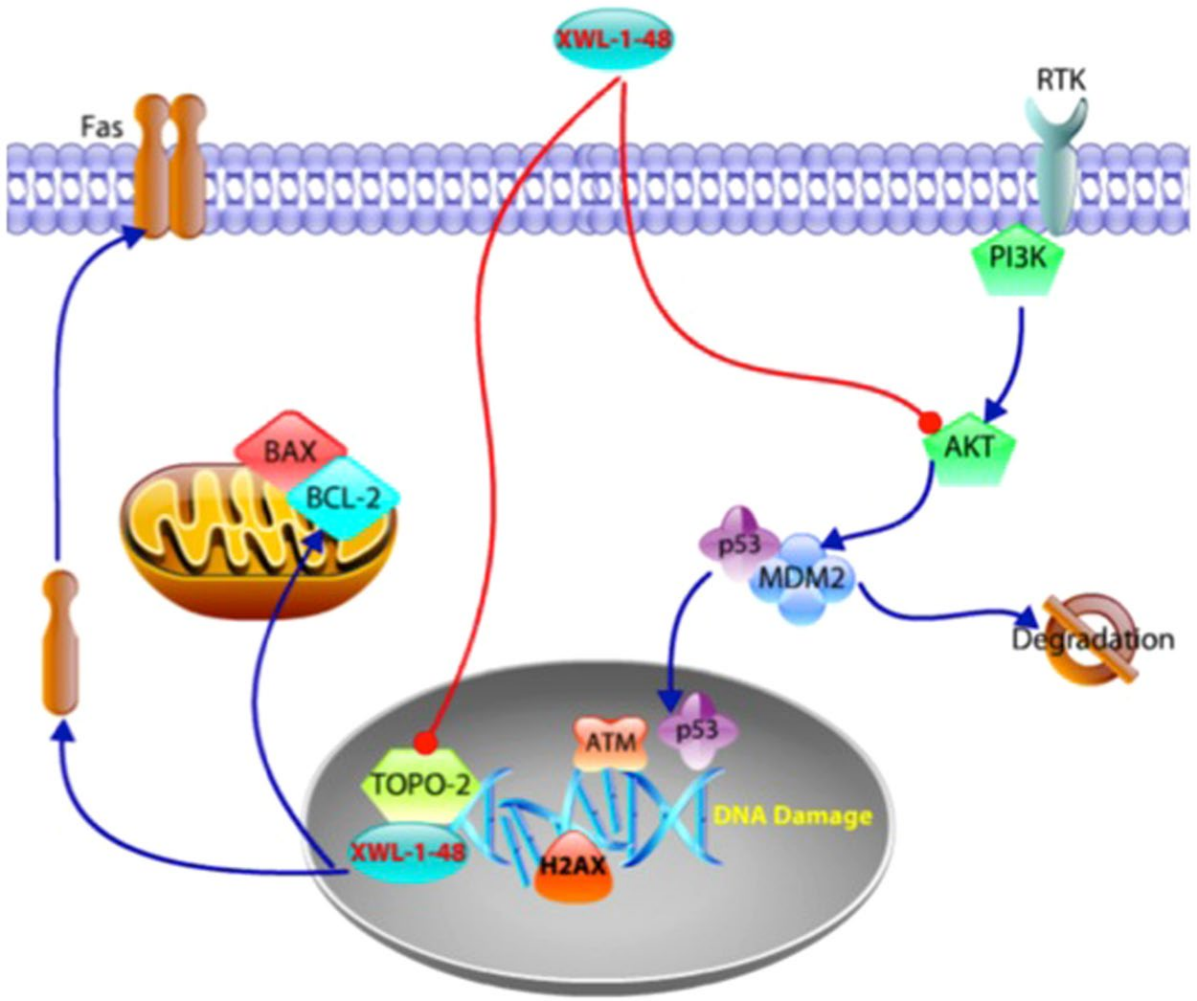
Signaling pathways altered by XWL-1-48. XWL-1-48 is a novel orally topoisomerase II inhibitor and exerts potent *in vitro* and in *vivo* antitumor activity against HCC. Treatment of XWL-1-48 significantly inhibited topoisomerase II activity, activated ATM/Chk2/Cdc25A and ATM/p53/p21 pathways, triggered DNA damage response, arrested cell cycle at S phase, and induced mitochondria- and death receptor-mediated apoptosis. Meanwhile, XWL-1-48 strongly blocked PI3K/Akt/Mdm2 pathway, enhanced degradation of Mdm2, and suppressed HCC cell survival.

In summary, our findings suggest that XWL-1-48 is a novel orally topoisomerase II inhibitor and exerts potent *in vitro* and in *vivo* anti-tumor activity in our HCC model. Treatment of XWL-1-48 significantly inhibited topoisomerase II activity, triggered DNA damage response, arrested cell cycle at S phase, and induced mitochondria- and death receptor-mediated apoptosis. Meanwhile, XWL-1-48 strongly blocked PI3K/Akt/Mdm2 pathway, enhanced degradation of Mdm2, and suppressed HCC cell survival (Fig. 8). Thus, XWL-1-48 may be a promising orally topoisomerase II inhibitor for treatment of HCC.

## Materials and Methods

### Drugs and chemicals

XWL-1-48, a new derivative of podophyllotoxin, was synthesized and identified by Dr. Xiao’s lab. The purity of XWL-1-48 is more than 98%, and it is freshly dissolved in dimethyl suifoxide (DMSO) before use. The final concentration of DMSO is less than 0.1% in all the experiments. The chemical structures of podophyllotoxin, XWL-1-48 and GL331 are shown in Fig. 1A-C. LY294002 (a pan-PI3K inhibitor), 1-(4, 5-dimethylthiazol-2-yl)-3, 5-diphenyformazan (MTT) and other chemicals were purchased from Sigma chemical Co. (St. Louis, MO).

### Cell lines and cell culture

HepG2 and MCF-7 cells were grown in DMEM (GIBCO) medium and other types of cancer cell were all grown in RPMI1640 (GIBCO) medium supplemented with 10% heat-inactivated newborn calf serum, 100 U/mL penicillin, and 100 µg/mL streptomycin. All cell lines were kept in our lab. All protocols using human cell lines were approved by the Research Ethics Committee of the Institute of Materia Medica, Chinese Academy of Medical Sciences & Peking Union Medical College, and conducted in accordance with the approved guidelines for safety requirements.

### kDNA-decatenation assay

kDNA-decatenation assay was preformed as previously described. In brief, the standard reaction mixture consisted of assay buffer (3 μL of 10xbuffer per assay), ATP (1 μL), kDNA (200ng) and water. 3 μL of XWL-1-48 or 0.1%DMSO was added into the reaction mixture. After that, 3 μL enzyme was added and incubated for 30 min at 37 °C, the reaction was stopped by adding 30 μL of STEB and 30 μL of chloroform/isoamyl alcohol (v:v, 24:1). The result was detected by agarose gel analysis.

### Cell viability assay

Cell viability was determined by MTT assay. As our previous described^33^, cells were seeded in 96-well plates, and then incubated with various concentrations of XWL-1-48 for additional 72 h. After that, 100 µL of 0.5 µg/mL MTT was added into each well after withdraw the culture medium and incubated for 4 h. The resulting formazan was dissolved in 150 µL DMSO after aspiration of the culture medium. Plates were shook for 30 min and read at 570 nm using a micro-plate reader (Bio-Rad Model 450). The IC_50_ was measured in duplicates and each experiment was repeated 3-5 times. IC_50_ value was calculated by Graphpad Prism 6.0 software.

### Clonogenic assay

HepG2 cells were plated in 6-well plates at density of 200 cells/well. On the following day, cells were incubated to XWL-1-48 (1, 2.5, 5 nM) or GL331 (5 nM) for 24 h. Then, the growth medium was changed to fresh medium. After 10-14 days incubation, colonies were fixed with trypan blue solution (75% methanol/25% acetic acid/0.25% trypan blue) for 15 min, rinsed with PBS twice, and air-dried before counting colonies >50 cells^19^.

### Cell cycle analysis

HepG2 cells were seeded into 6-well plates at density of 4.0 × 10^5^. After exposed to XWL-1-48 (0.1, 0.3, 1 µM) or GL331 (1 µM) for 24 h, the cells were harvested, fixed in 70% ice-cold ethanol, and stored at 4 °C overnight. After that, cells were washed and transferred into PBS, incubated with RNase A (50 mg/mL) for 30 min at 37 °C, followed by 30 min treatment with propidium iodide (PI, 50 mg/mL) at 37 °C. The cells were resuspended in PBS. The effect of XWL-1-48 and GL-331 on cell cycle were analyzed by flow cytometry (Beckman Coulter, USA).

### AnnexinV-propidium iodide binding assay

HepG2 cells were incubated with XWL-1-48 (0.1, 0.3, 1 µM) or GL331 (1 µM) for 24 h, and then resuspended with the cold binding buffer. 5 µL of Annexin-V-FITC and 5 µL of PI were added and the cells were further incubated for 10 min in dark at room temperature. Apoptotic cells were analyzed by flow cytometry (Beckman coulter, USA).

### Caspase-3 colorimetric assay

The ability of XWL-1-48 or GL331 on caspase-3 activity was measured by caspase-3 colorimetric assay Kit (KeyGEN Biotech Inc., China). After exposure to XWL-1-48 or GL331 for 24 h, HepG2 cells were harvested and lysed. 100 μg protein in 90 μL assay buffer were mixed with 10 μL caspase-3 specific synthetic fluorescent substrates. After incubation for 4 h at 37 °C, yellowish color from the pNA was read at 405 nm on Fluorescence Reader (Bio-TEK INSTRUMENTS Inc., USA).

### RNA interference

HepG2 cells were seeded into 6-well plate at concentration of 2.0 × 10^5^ cells/well. On the following day, specific siRNA targeting to p53 (GAGGUUGGCUCUGACUGUA, Dharmacon; Chicago, IL) or to ATM (5′-CUA ACA AAC AGG UGA UAU AUU-3′, Dharmacon; Chicago, IL) was mixed with Lipofectamine 2000 in serum-free DMEM medium and transfected to the plated cells. After 24 h transfection, cells were treated with XWL-1-48 (1 µM) for another 24 h.

### RT-PCR analysis

After cultured with XWL-1-48 (0.1, 0.3, 1 µM, respectively) for 24 h, HepG2 cells were harvested. According to the guanidine isothiocyanate/phenol/chloroform method, the Total RNA was extracted. The integrity and purity of the RNA were evaluated by UV Spectrophotometer for OD260 and OD280, then reverse transcripted from mRNA to cDNA using the RT-PCR kit (Promega, WI, USA). The following primers were used for RT-PCR: MDM2 (509 bp) sense 5′-AGA AGG TTC TGG GAA GAT CGC-3′, anti-sense 5′-GTT GAT GGC TGA GAA TAG-3′; β-actin (529 bp) sense 5′-CTT GAT GCT GGT GTA AGT-3′, anti-sense 5′-AGC ACT GTG TTG GCG TAC AG-3′. The PCR profile was as follows: 10 min at 95 °C, followed by 30 cycles of 30 s at 95 °C and 1 min at 60 °C. The PCR product was detected by 1% agarose gel electrophoresis with ethidium bromide staining and viewed by UV transillumination.

### Western blot analysis

Cells were rinsed with PBS, and then harvested, lyzed in lysis buffer (Applygen Technologies Inc. China) for 30 min on ice, centrifuged 12000 g for 20 min at 4 °C. Protein concentrations were measured by BCA assay. 30 µg of protein of each group was resolved by 10% SDS-PAGE, and further transferred to PVDF membrane. The membrane was blocked in 5% fat-free milk with TBST for 1 h at room temperature. The membrane was incubated with anti-γ**-**H2AX, anti-p21, anti-p-ATM, anti-ATM, anti-p-Chk2, anti-Chk2, anti-Cdc25A, anti-p-p53, anti-AKT, anti-active caspase-3 and anti-caspase-3 (Cell Signaling Technology, USA), anti-p53, anti-Bax, anti-Bcl-2, anti-Fas, anti-Fas L anti-α-tubulin (Santa Cruz, USA) antibodies in 5% milk TBST, at 4 °C overnight. After washed 3 times, the membrane were incubated with secondary antibodies for 1 h. Then, membranes were washed and subsequently developed using ECL (FujiFilm, Japan) reagent (Applygen Technologies Inc. China). All immnuoblot images were quantified by Gel-Pro analyzer4.0 software.

### Immunoprecipitation analysis

HepG2 cells were lyzed in NET buffer (Applygen Technologies Inc. China) on ice for 30 min, and then centrifuged 12,000 g for 30 min at 4 °C. 2 mg protein of each group were immunoprecipitated with Mdm2 antibody (1 µg) by overnight incubations at 4 °C after adjusting the volumes to 1.0 mL with cold NET buffer. The immune complexes were precipitated with Protein-A Sepharose CL-4B (GE Healthcare Inc. USA) and washed three times with TNT buffer (Applygen Technologies Inc. China), once with NET buffer and once with PBS. Immunoprecipitated proteins were determined by Western blot assay using anti-ubiquitin polyclonal antibody^24^.

### Immunofluorescence microscopy

HepG2 cells were plated onto poly-L-lysine coated cover slips in 6-well plates. For analysis, the cells subjected to different treatments were fixed with 4% paraformaldehyde at room temperature for 15 min, washed three times with PBS and permeabilized in PBS containing 0.1% Triton X-100 for 10 min. The cover slips were washed three times with PBS again and then blocked in 3% normal goat serum for 2 h with shaking. The cells were counter-stained with DAPI. Cover slips were then washed three times with PBS mounted onto slides using fluorescent mounting medium. Intensity changes in the γ-H2AX and p-ATM were imaged with an Olympus FV1000 (Olympus, Tokyo, Japan).

### Xenograft mouse model

All animal experiments were approved by the Ethics Committee for Animal Experiments of the Institute of Materia Medica, Chinese Academy of Medical Sciences & Peking Union Medical College and conducted in accordance with the Guidelines for Animal Experiments of Peking Union Medical College. The female BALB/c nude mice (Center of Experimental Animals, Chinese Academy of Medical Sciences; 6-7 weeks of age and body weight of 18-20 g) were implanted with 5 × 10^6^ HepG2 cells by subcutaneous injection into the interscapular area. Xenografts were maintained for two generations by subcutaneous implantation of about 50 mg non-necrotic tumor tissues using a trocar^34^. According to the formula, V = 1/2ab^2^, in which “a” and “b” represents length and width of tumor in mm, the tumor volume was calculated. Drug treatment was started when the tumor size reached to above 100 mm^3^. The xenograft mice were divided into 4 groups randomly, and 7 mice per group. VP16 (23 mg/kg) as positive control was administered on day 1 after grouping the mice and then every other day for one time. Sterile normal saline-treated group used as normal control. XWL-1-48 (2 and 4 mg/kg) dissolved in sterile normal saline was orally administrated (o.p) in a volume of 0.2 ml/20 g body weight from day 1, then every other day for 2 weeks. The tumor growth curve was drawn. At the end point of experiment, tumors were excised from the mice and weighted it and then snap frozen in liquid nitrogen and stored at -80 °C. The rate of inhibition (IR) was also calculated.

### Statistical analysis

Data are presented as mean ± SD. Statistical analysis of the data was performed using the one-way ANOVA by SPSS software. *p* < 0.05 was considered statistically significant.
